# Supplementary figures and images for: Pp6-Pfkfb1 axis modulates intracellular bacterial proliferation by orchestrating host-pathogen metabolic crosstalk
Source: PLoS Pathog. 2025 Dec 31;21(12):e1013304. doi: 10.1371/journal.ppat.1013304 (PMC12782431; doi:10.1371/journal.ppat.1013304)

**
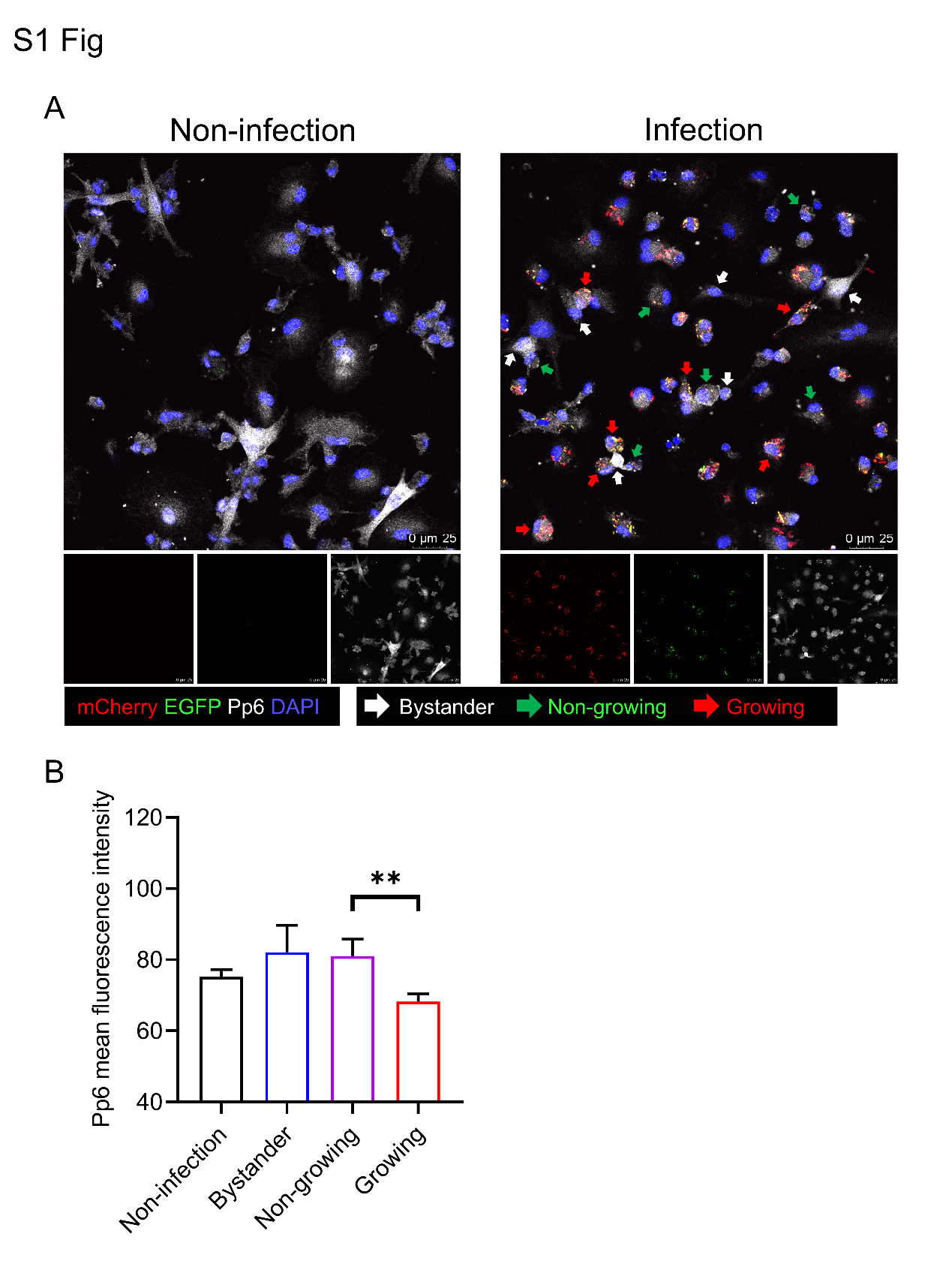
**

**S1 Fig. The expression of Pp6 in BMDMs after infection (unsorted)**

Supplement: S1 Fig — (A-B) The expression of Pp6 in BMDMs after infection (unsorted) was detected by immunocytochemistry (A) and statistical data were shown in (B). Scale bar, 25 μm. P-values were determined by two-tailed unpaired t-test (mean ± SEM). **P < 0.01. (DOCX) [file ppat.1013304.s001.docx]

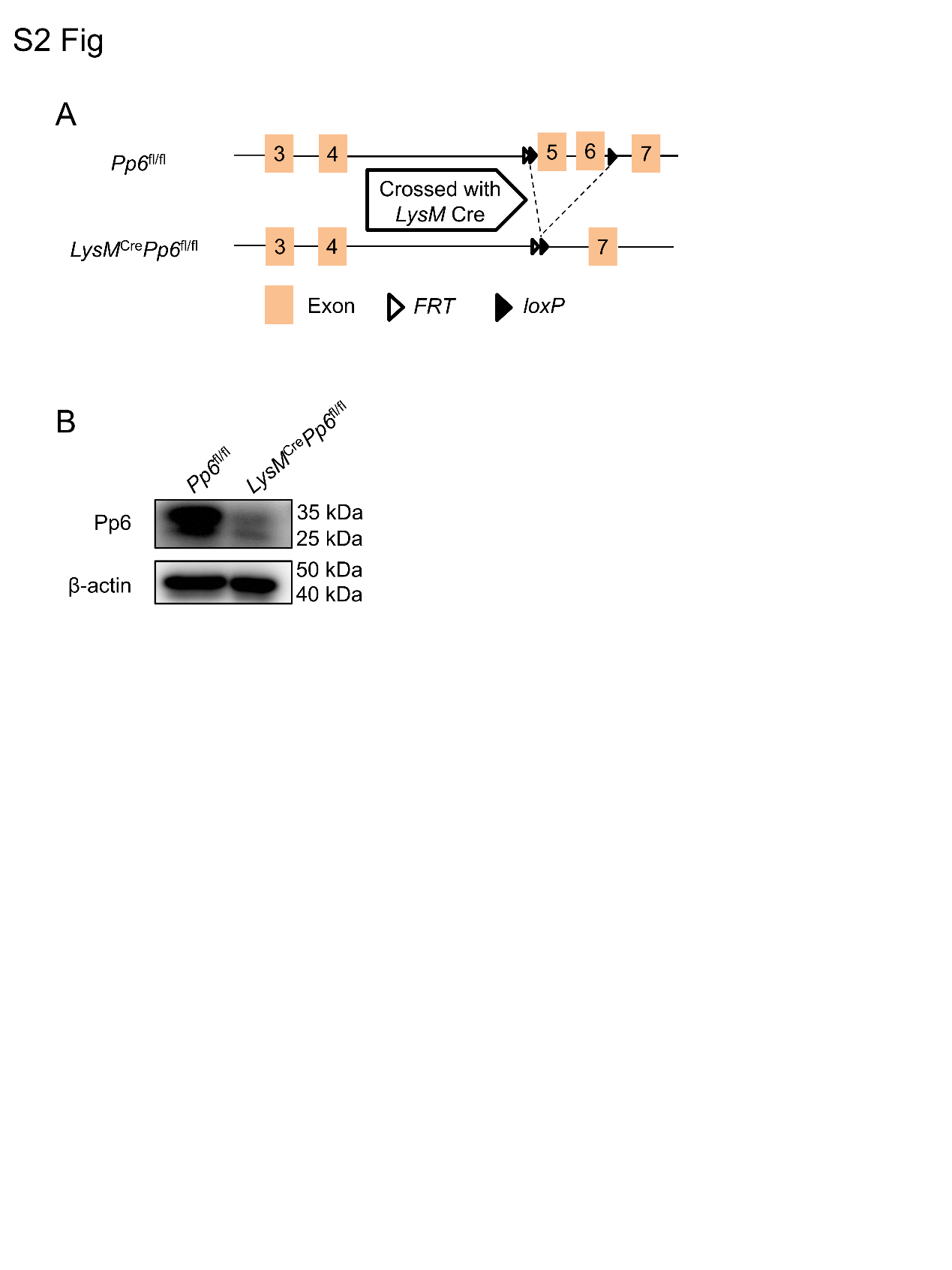


**S2 Fig. Strategies for *Pp6* conditional knockout mouse construction**

Supplement: S2 Fig — (A-B) Schematic of Pp6 deletion in LysMCrePp6fl/fl mice (A) and western blot analysis of Pp6 expression in BMDMs from LysMCrePp6fl/fl and Pp6fl/fl mice (B). (DOCX) [file ppat.1013304.s002.docx]

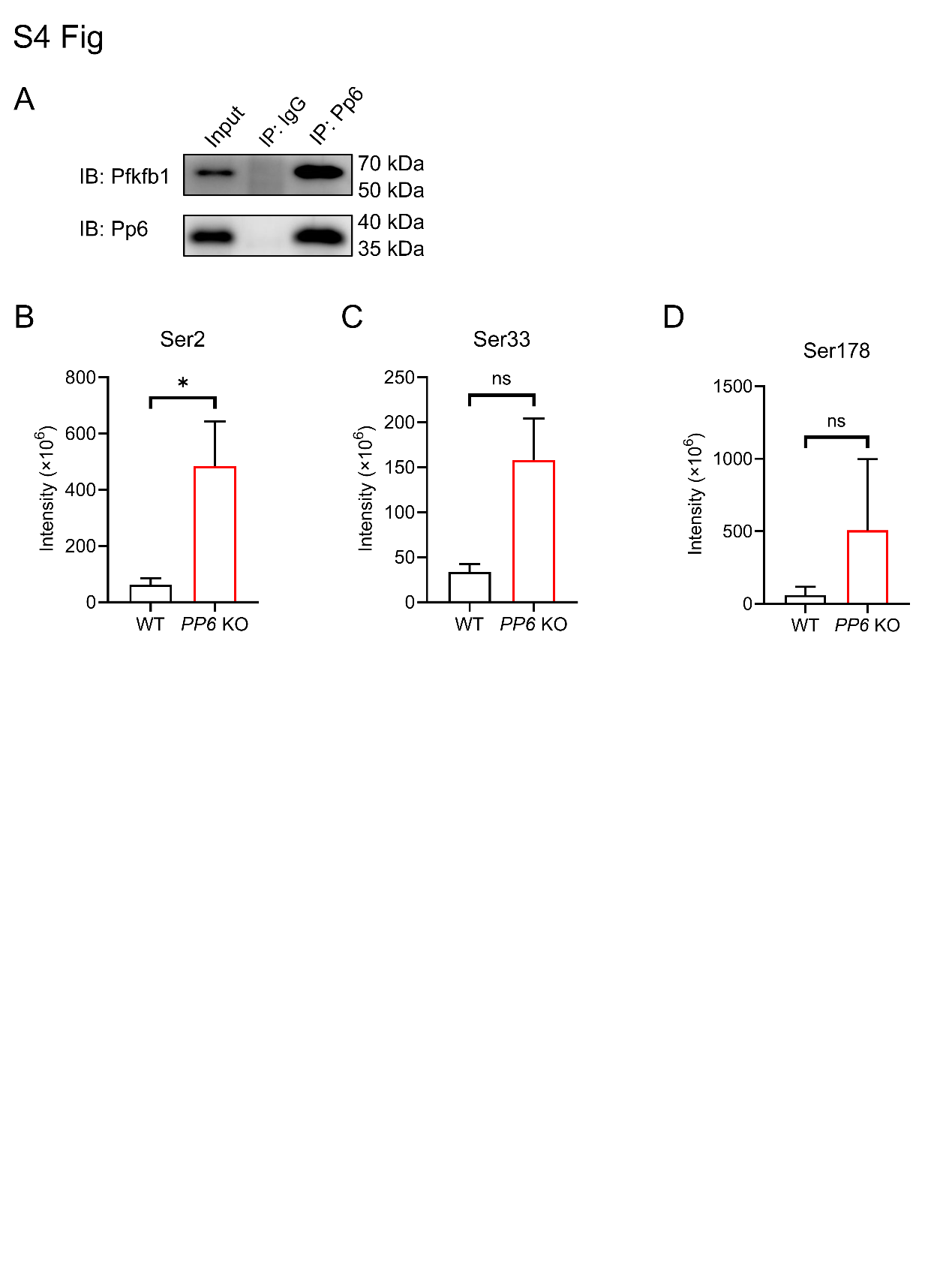


**S4 Fig. Ser-2, Ser-33 and Ser-178 sites were hyper-phosphorylated in *PP6*-deficient 293FT cells**

Supplement: S4 Fig — (A) Lysates from Salmonella-infected BMDMs were immunoprecipitated with IgG or anti-PP6C, and blotted with anti-PFKFB1 and anti-PP6C. (B-D) LC-MS/MS-based detection of phosphorylation sites on PFKFB1 immunoprecipitates from WT and PP6-deficient 293FT cells. Intensity of Ser-2 (B), Ser-33 (C), Ser-178 (D) in WT and PP6-deficient 293FT cells. P-values were determined by two-tailed unpaired t-test (mean ± SEM). ns: not significant, *P < 0.05. (DOCX) [file ppat.1013304.s004.docx]

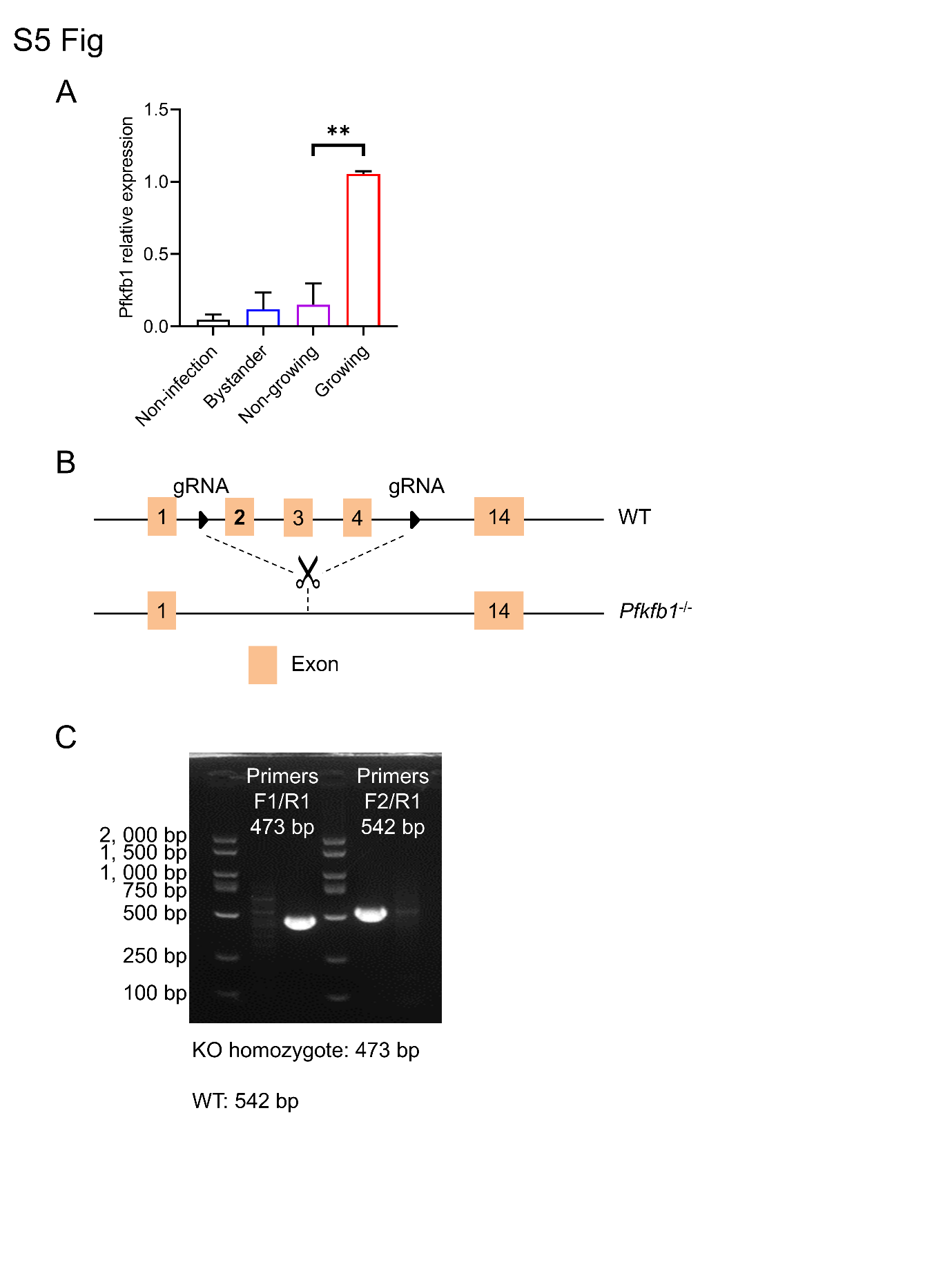


**S5 Fig. Strategies for *Pfkfb1* knockout mouse construction**

Supplement: S5 Fig — (A) Quantification of Pfkfb1 in non-infected BMDMs, bystander BMDMs and BMDMs containing growing and non-growing bacteria (n = 3). P-value was determined by two-tailed unpaired t-test (mean ± SEM). **P < 0.01. (B-C) Schematic of Pfkfb1 deletion in Pfkfb1-/- mice (B) and Pfkfb1 genotyping of the toes derived from WT and Pfkfb1-/- mice (C). (DOCX) [file ppat.1013304.s005.docx]
