# Supplementary material for: Pp6-Pfkfb1 axis modulates intracellular bacterial proliferation by orchestrating host-pathogen metabolic crosstalk
Source: PLoS Pathog. 2025 Dec 31;21(12):e1013304. doi: 10.1371/journal.ppat.1013304 (PMC12782431; doi:10.1371/journal.ppat.1013304)
Supplement: S3 Fig — (A) Luciferase activity assessment in BMDMs transfected with miR-31 mimics and either the WT or point-mutated 3’ UTR reporter construct (n = 4). P-values were determined by two-tailed unpaired t-test (mean ± SEM). ns, not significant, ***P < 0.001. (B) Quantification of Pp6 relative expression in BMDMs from WT and miR-31-/- mice (n = 4). P-value was determined by two-tailed unpaired t-test (mean ± SEM). *P < 0.05, **P < 0.01. (C-D) Schematic of miR-31 deletion in LysMCremiR-31fl/fl mice (C) and western blot analysis of Pp6 expression in BMDMs from LysMCremiR-31fl/fl and miR-31fl/fl mice (D). (DOCX) [file ppat.1013304.s003.docx]

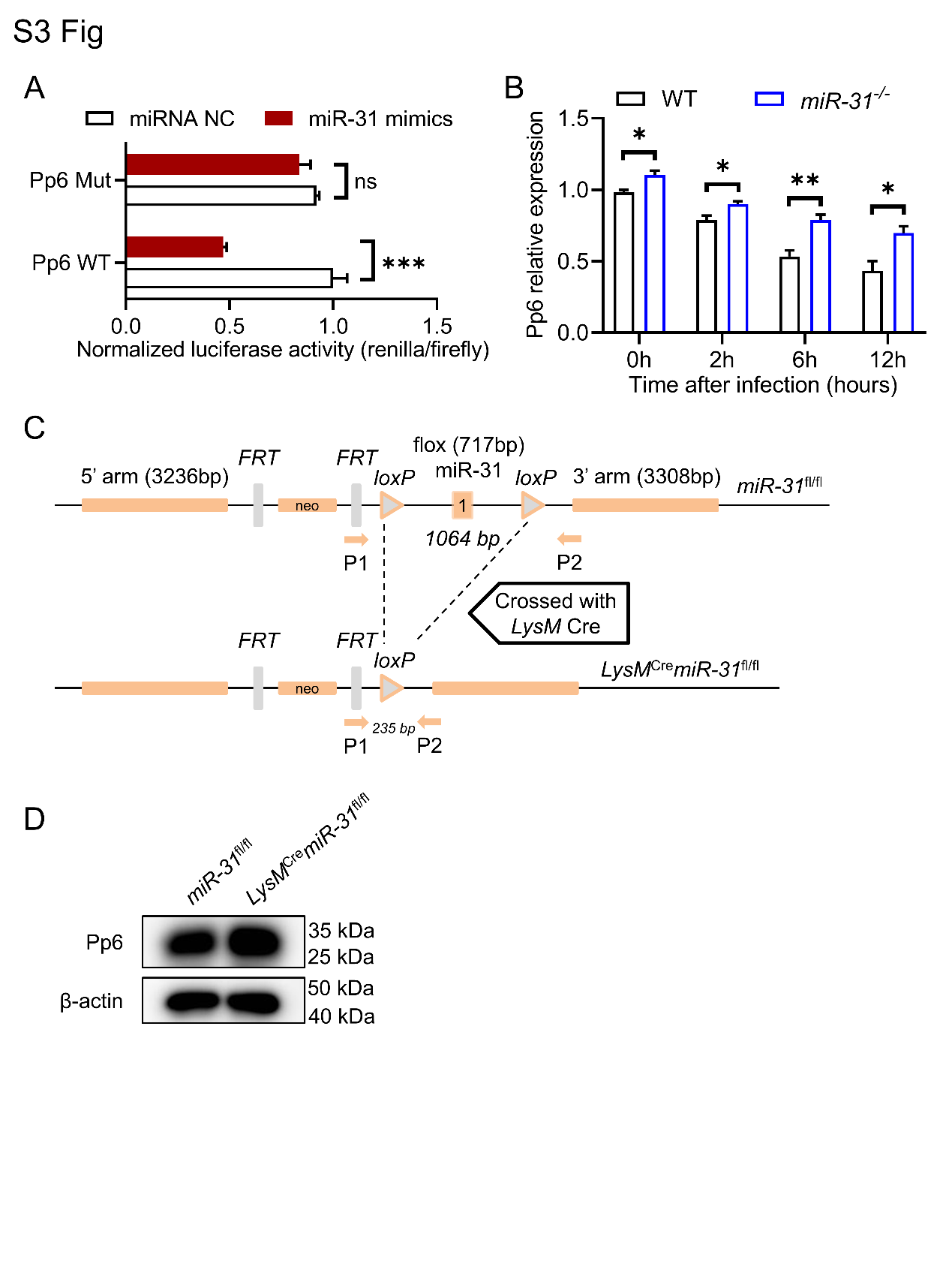


**S3 Fig. miR-31 directly targets Pp6 in BMDMs and strategies for *miR-31* conditional knockout mouse construction**
